# High-Risk International Clones of Carbapenem-Nonsusceptible Pseudomonas aeruginosa Endemic to Indonesian Intensive Care Units: Impact of a Multifaceted Infection Control Intervention Analyzed at the Genomic Level

**DOI:** 10.1128/mBio.02384-19

**Published:** 2019-11-12

**Authors:** Andreu Coello Pelegrin, Yulia Rosa Saharman, Aurélien Griffon, Mattia Palmieri, Caroline Mirande, Anis Karuniawati, Rudyanto Sedono, Dita Aditianingsih, Wil H. F. Goessens, Alex van Belkum, Henri A. Verbrugh, Corné H. W. Klaassen, Juliëtte A. Severin

**Affiliations:** a Clinical Unit, bioMérieux, La Balme Les Grottes, France; b Vaccine & Infectious Disease Institute, Laboratory of Medical Microbiology, Faculty of Medicine and Health Sciences, University of Antwerp, Antwerp, Belgium; c Department of Clinical Microbiology, Faculty of Medicine, Universitas Indonesia/Dr. Cipto Mangunkusumo General Hospital, Jakarta, Indonesia; d Department of Medical Microbiology and Infectious Diseases, Erasmus MC University Medical Center, Rotterdam, The Netherlands; e R&D Systems & Development, bioMérieux, Marcy l’Etoile, France; f Microbiology R&D, bioMérieux, La Balme Les Grottes, France; g Critical Care Division, Department of Anesthesia and Intensive Care, Faculty of Medicine, Universitas Indonesia/Dr. Cipto Mangunkusumo General Hospital, Jakarta, Indonesia; UNC Health Care System

**Keywords:** *Pseudomonas aeruginosa*, intensive care units, infection control, single nucleotide polymorphism, Indonesia, microbial drug resistance

## Abstract

Infection control effectiveness evaluations require detailed epidemiological and microbiological data. We analyzed the genomic profiles of carbapenem-nonsusceptible Pseudomonas aeruginosa (CNPA) strains collected from two intensive care units (ICUs) in the national referral hospital in Jakarta, Indonesia, where a multifaceted infection control intervention was applied. We used clinical data combined with whole-genome sequencing (WGS) of systematically collected CNPA to infer the transmission dynamics of CNPA strains and to characterize their resistome. We found that the number of CNPA transmissions and acquisitions by patients was highly variable over time but that, overall, the rates were not significantly reduced by the intervention. Environmental sources were involved in these transmissions and acquisitions. Four high-risk international CNPA clones (ST235, ST823, ST375, and ST446) dominated, but the distribution of these clones changed significantly after the intervention was implemented. Using resistome analysis, carbapenem resistance was explained by the presence of various carbapenemase-encoding genes (*bla*_GES-5_, *bla*_VIM-2-8_, and *bla*_IMP-1-7-43_) and by mutations within the porin OprD. Our results reveal for the first time the dynamics of P. aeruginosa antimicrobial resistance (AMR) profiles in Indonesia and additionally show the utility of WGS in combination with clinical data to evaluate the impact of an infection control intervention. (This study has been registered at www.trialregister.nl under registration no. NTR5541).

## Abstract (precis)

In low-to-middle-income countries such as Indonesia, work in intensive care units (ICUs) can be hampered by lack of resources. Conducting large epidemiological studies in such settings using genomic tools is rather challenging. Still, we were able to systematically study the transmissions of carbapenem-nonsusceptible strains of P. aeruginosa (CNPA) within and between ICUs, before and after an infection control intervention. Our data show the importance of the broad dissemination of the internationally recognized CNPA clones, the relevance of environmental reservoirs, and the mixed effects of the implemented intervention; it led to a profound change in the clonal make-up of CNPA, but it did not reduce the patients’ risk of CNPA acquisitions. Thus, CNPA epidemiology in Indonesian ICUs is part of a global expansion of multiple CNPA clones that remains difficult to control by infection prevention measures.

## INTRODUCTION

Pseudomonas aeruginosa is especially dreaded as one of the leading species causing health care-associated infections (1, 2). P. aeruginosa is an opportunistic human pathogen with a remarkably versatile genome, which allows it to adapt to a wide range of environments and conditions and, consequently, to survive in a variety of niches. This is mainly due to traits encoded in its accessory genome, which includes genes coding for antimicrobial resistance (AMR), a great diversity of metabolic pathways, and virulence factors (3). AMR is a major concern in clinical P. aeruginosa isolates, as almost 31% of all invasive isolates are resistant to at least one of the main antimicrobial groups tested, according to the most recent AMR surveillance report by the European Centre for Disease Prevention and Control (ECDC) (4). Additionally, a limited number of P. aeruginosa clones with multidrug resistance (MDR) profiles are particularly worrisome since they have been shown to have achieved nearly global expansion (5, 6). Especially in low-to-middle-income countries, MDR P. aeruginosa contributes to in-hospital mortality (7, 8). Gathering as much clinical and microbiological information as possible with respect to these isolates is essential to inform nosocomial infection control and surveillance procedures.

During recent years, whole-genome sequencing (WGS) has developed rapidly into a reference tool for outbreak management (9–12). However, there is not a common standardized and accepted methodology to infer bacterial transmissions during outbreak investigations from WGS data. This is troublesome, especially when the WGS approach is implemented in regions of the world where MDR and extensively drug-resistant (XDR) microorganisms are already endemic.

The aim of this study was to assess nosocomial transmission of carbapenem-nonsusceptible P. aeruginosa (CNPA) using WGS in combination with detailed clinical data from intensive care unit (ICU) patients of the national referral hospital of Indonesia. CNPA isolates were systematically collected before and after an infection control intervention so that we could study its effect on the dynamics of transmission of CNPA in this setting in detail. Additionally, we highlight the main P. aeruginosa clones found as well as their resistomes. Risk factors for the carriage and acquisition of CNPA and its effect on patients’ outcomes have been analyzed and published separately (13).

## RESULTS

A total of 412 patients were included in the study during the preintervention phase (188 were admitted to the adult ICU and 224 to the emergency room ICU [ER-ICU]), and at least one CNPA strain was isolated from 51 (12.4%) patients. A total of 363 patients (adult ICU, 133; ER-ICU, 230) were included during the postintervention phase, and at least one CNPA strain was isolated from 52 (14.3%) patients (8). Risk factors, including antibiotic usage, and patient outcomes of CNPA carriage and acquisition during ICU stay are reported elsewhere (13). A total of 119 CNPA strains were isolated during the preintervention phase, here defined as the “preintervention phase” set, which included 12 environmental isolates. 118 CNPA strains were isolated during the postintervention phase, here named the “postintervention phase” set, including 3 environmental isolates. Note that the two strain collections contained multiple CNPA isolates from 51 patients. In order to avoid overrepresentation of specific genotypes, in the indicated calculations we used only the first isolate per unique genotype per patient (see below and Table S3).

### Phenotypic identification and antibiotic susceptibility testing (AST) of the P. aeruginosa strains.

Vitek mass spectrometry (MS) confirmed the correct identification of all P. aeruginosa isolates (data not shown). A total of 130/237 (54.9%) isolates were resistant to all antibiotics tested. Details of the results of susceptibility testing are presented at the isolate level in Table S1 in the supplemental material.

10.1128/mBio.02384-19.3 TABLE S1 Detailed antibiotic susceptibility testing performed by Vitek 2 of the 237 isolates. Categories of isolates are indicated as follows: Y (yes), isolates that appear in Results in the article; N (no), isolates excluded from description because of genotype overrepresentation. Download Table S1, XLSX file, 0.02 MB. Copyright © 2019 Pelegrin et al. 2019 Pelegrin et al. This content is distributed under the terms of the Creative Commons Attribution 4.0 International license.

### Sequencing statistics and assembly quality.

The median *N*_50_ value was 225,489 bp (interquartile range [IQR], 195,941 to 269,325 bp), and the median number of contigs was 114 (IQR, 72 to 148). Genomes sizes ranged from 6.3 Mb to 7.3 Mb. FastANI identity values for all assemblies were over 98.5%, confirming the correct species identification of all isolates. Detailed sequencing statistics and QUAST (14) results are summarized in Table S2. The quality criteria were met for all sequences.

10.1128/mBio.02384-19.4 TABLE S2 Whole-genome sequencing quality control parameters. *, average nucleotide identity analysis: reference Pseudomonas aeruginosa PAO1 (GenBank accession no. NC_002516.2). Download Table S2, XLSX file, 0.04 MB. Copyright © 2019 Pelegrin et al. 2019 Pelegrin et al. This content is distributed under the terms of the Creative Commons Attribution 4.0 International license.

### *In silico* MLST.

**FIG 1:**
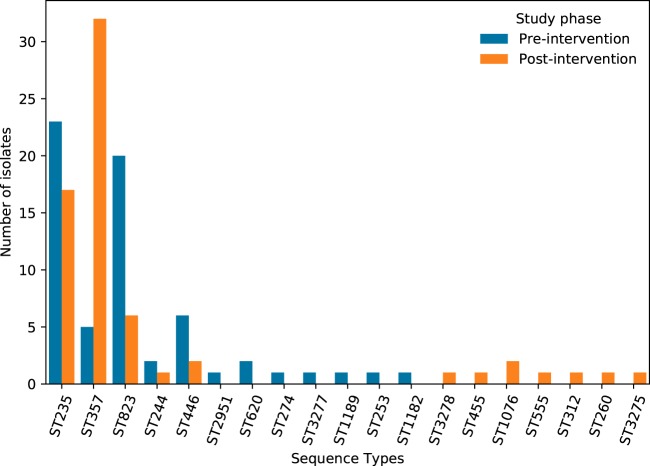
Multilocus sequence type of genotype-corrected CNPA. Sequence types are displayed in the abscissa axis. The ordinate axis indicates the number of genotype-corrected isolates.

We identified four major CNPA sequence types (STs) (ST235, ST357, ST823, and ST446) and three new sequence types (ST3275, ST3277, and ST3278) along with 12 minor STs (Fig. 1). A goeBURST analysis showed that 17/19 of the sequence types found belonged to an already-existing P. aeruginosa clonal complex, while ST1189 and ST3277 were singletons (see Fig. S1 in the supplemental material). We observed a clear shift in the sequence type distribution between the two phases of the study: while ST235 was the dominant sequence type in the preintervention phase, ST357 emerged as the dominant ST in the postintervention phase. Only the main sequence types and ST244 were present in both phases of the study; the remainder were detected only in the preintervention phase (ST2951, ST620, ST274, ST1189, ST253, ST3277, and ST1182) or only in the postintervention phase (ST3278, ST455, ST1076, ST555, ST312, ST260, and ST3275). Detailed data regarding the MLST profiles of all isolates are provided in Table S3.

10.1128/mBio.02384-19.1 FIG S1 goeBURST of the 3,321 sequence types listed in the Pseudomonas aeruginosa PubMLST database (August 2019). Blue points represent sequence types (STs); lines connect single-locus variants (SLVs). Red boxes within the circles are the STs found in this study; light green boxes represent the clonal complex group founder and dark green boxes the subgroup founder. ST1189 and ST3277 are singletons. Download FIG S1, PDF file, 0.7 MB. Copyright © 2019 Pelegrin et al. 2019 Pelegrin et al. This content is distributed under the terms of the Creative Commons Attribution 4.0 International license.

10.1128/mBio.02384-19.5 TABLE S3 Detailed results of multilocus sequence typing of the 237 isolates. Download Table S3, DOC file, 0.05 MB. Copyright © 2019 Pelegrin et al. 2019 Pelegrin et al. This content is distributed under the terms of the Creative Commons Attribution 4.0 International license.

### Analysis of AMR determinants.

**FIG 2:**
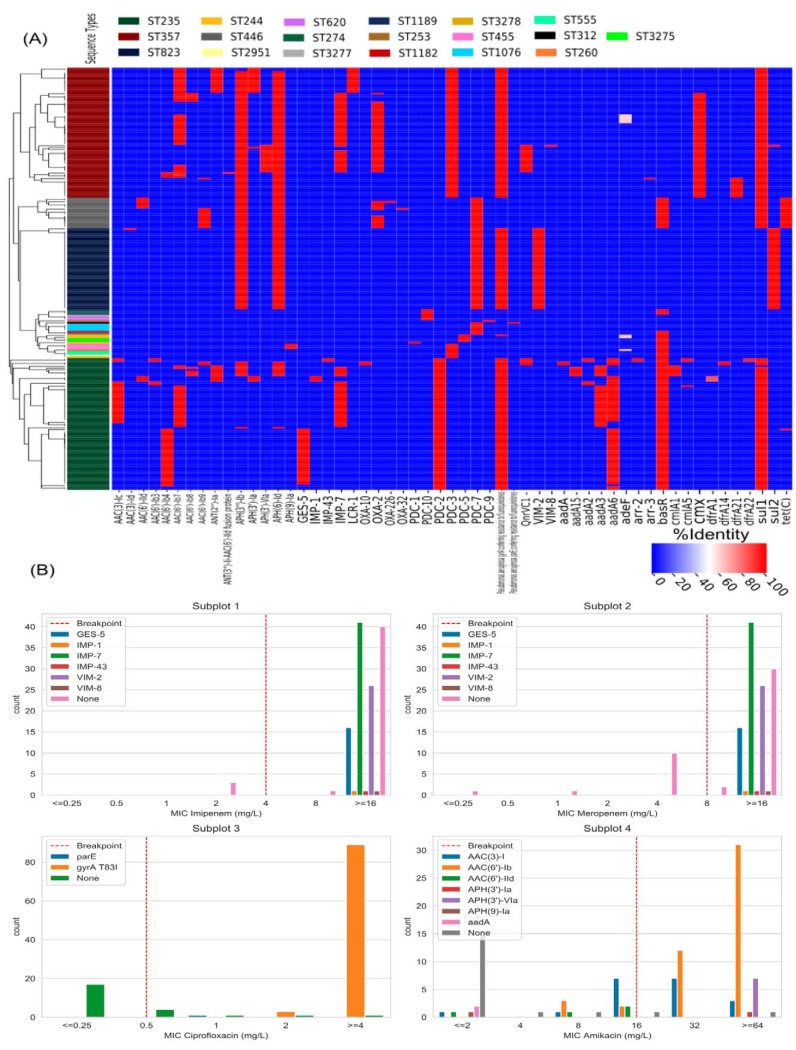
(A) Antimicrobial resistance heat map of 130 isolates of carbapenem-nonsusceptible P. aeruginosa (CNPA). The *x* axis contains only AMR determinants that were variably present in the CNPA collection. The following AMR determinants are not displayed because they were present in all isolates: *APH(3′)-IIb* (aminoglycoside resistance); *bla*_OXA-50_ (β-lactam resistance); *fosA* (fosfomycin resistance); *bcr-1* (bicyclomycin resistance); *arnA* and *basS* (polymyxin resistance); *catB* (chloramphenicol resistance); *pmpM* (multidrug and toxic compound extrusion [MATE] transporter); *emrE* (small multidrug resistance efflux pump); *crpP* (quinolone resistance); *mexA-mexB-oprM* plus *mexR*, *nalC*, and *nalD* plus *cpxR* plus ArmR (resistance-nodulation-cell division [RND] efflux pump plus *mexAB* repressors plus *mexAB* activator plus *mexR* inhibitor); *mexC-mexD-oprJ* plus NfxB (RND efflux pump plus *mexCD*-*oprJ* repressor); *mexE-mexF-oprN* plus *mexT* plus *mexS* (RND efflux pump plus *mexEF* activator plus *mexT* suppressor); *mexG-mexH-mexI-opmD* plus *soxR* (RND efflux pump plus transcriptional activator); *mexJ-mexK-opmH* plus *mexL* (RND efflux pump plus mexJK repressor); *mexM-mexN-oprM* (RND efflux pump); *mexP-mexQ-opmE* (RND efflux pump); *mexV-mexW-oprM* (RND efflux pump); *muxA-muxB-muxC-opmB* (RND efflux pump); *triA-triB-triC-opmH* (RND efflux pump); *mexY* plus *mexZ* (RND efflux pump component plus *mexXY* transcriptional regulator). (B) Bar plots showing the relation between the MIC of imipenem, meropenem, ciprofloxacin, and amikacin (subplots 1 to 4) and their related resistance genes found among the CNPA according to the literature. Vertical red dashed lines mark the EUCAST 2019 resistance breakpoints.

We found 102 different AMR-related genes, among which at least 32 represented acquired resistance genes according to the literature. Additionally, 70 genes were analyzed with snippy, which brought to light a considerable amount of mutations directly related to antibiotic resistance proteins (such as the AmpC cephalosporinase or penicillin-binding proteins) and mutations in intrinsic genes such as resistance-nodulation-cell division (RND) efflux pumps and regulators. The mutational resistome obtained with snippy can be found in Table S4. Panel A of Fig. 2 presents a heat map of the AMR determinants found by the use of the Resistance Gene Identifier-Comprehensive Antibiotic Resistance Database (RGI-CARD) in each genotypically unique strain in the CNPA collection. Eighteen beta-lactam resistance genes were detected, among them genes encoding the carbapenem-degrading enzymes *bla*_GES-5_ (16/130, 12.3%), *bla*_IMP-1_ (1/130, 0.8%), *bla*_IMP-7_ (42/130, 32.3%), *bla*_IMP-43_ (1/130, 0.8%), *bla*_VIM-2_ (26/130, 20.0%), and *bla*_VIM-8_ (1/130, 0.8%). Interestingly, some of these genes were restricted to certain CNPA clones, including the *bla*_GES-5_, *bla*_IMP-1_, *bla*_IMP-43_, *bla*_OXA-10_, and *bla*_VIM-8_ genes that were found only in ST235, while ST823 was the only sequence type harboring *bla*_VIM-2_. As expected, strains carrying these carbapenemase genes had high imipenem and meropenem MIC values (Fig. 2B, subplots 1 and 2).

10.1128/mBio.02384-19.6 TABLE S4 Direct and intrinsic mutational resistomes associated with antimicrobial resistance. “Direct mut. Resistome” indicates missense variations leading to amino acid point mutations in genes directly associated with antimicrobial resistance to β-lactams, aminoglycosides, quinolones, and/or polymyxins. “Intrinsic mut. resistome” indicates intrinsic genes (mainly RND efflux pumps determinants and regulators) with amino acid point mutations. Blank boxes signify that no missense variation was found. Download Table S4, XLSX file, 0.10 MB. Copyright © 2019 Pelegrin et al. 2019 Pelegrin et al. This content is distributed under the terms of the Creative Commons Attribution 4.0 International license.

The mutational analysis of OprD revealed 32 different missense mutations, including insertions leading to frameshifts (Fig. S2). Only one isolate had an OprD sequence identical to that of the type strain PAO1; the rest were found to have accumulated a pattern of point mutations that led to amino acid changes in the primary protein structure of the OprD porin. Eight of the 32 amino acid substitutions were present in more than 50% of the analyzed isolates, including the following: T_103_S (83/130, 63.9%), K_115_T (83/130, 63.9%), F_170_L (82/130, 63.0%), E_185_Q (118/130, 90.8%), P_186_G (116/130, 89.2%), V_189_T (118/130, 90.8%), R_310_E (92/130, 70.8%), and A_315_G (88/130, 67.7%). Details of the results of analyses of the amino acid substitution patterns can be found in Table S4. We observed that in carbapenemase-nonproducing strains, certain OprD types were prone to have higher imipenem and meropenem MIC values (Fig. 2B), but we could not establish a conclusive correlation between these porin gene mutations and the phenotypic susceptibility patterns of the CNPA strains.

10.1128/mBio.02384-19.2 FIG S2 OprD types among the carbapenemase-nonproducing strains. Three main OprD types and 11 other subtypes were observed. The point mutation pattern is described below. Note that subtypes *I.a*, *I.a2*, and *I.a3* additionally carried the _372_(VDSSSS-YAGL-)_383_ indel in the C-terminal part of OprD, first described by Epp et al. (35), as follows: for *I.a*, S_57_E, S_59_R, V_127_L, E_185_Q, P_186_G, V_189_T, E_202_Q, I_210_A, E_230_K, S_240_T, N_262_T, T_276_A, A_281_G, K_296_Q, Q_301_E, R_310_E, A_315_G, L_347_M, S_403_A, and Q_424_E; for *I.a2*, V_127_L, E_185_Q, P_186_G, V_189_T, E_202_Q, I_210_A, E_230_K, S_240_T, N_262_T, T_276_A, A_281_G, K_296_Q, Q_301_E, R_310_E, A_315_G, L_347_M, S_403_A, and Q_424_E; for *I.a3*, S_57_E, S_59_R, V_127_L, E_185_Q, P_186_G, V_189_T, E_202_Q, I_210_A, E_230_K, S_240_T, N_262_T, T_276_A, A_281_G, K_296_Q, Q_301_E, R_310_E, A_315_G, V_352_L, S_403_A, and Q_424_E; for *I.b*, D_43_N, S_57_E, S_59_R, V_127_L, E_185_Q, P_186_G, V_189_T, E_202_Q, I_210_A, E_230_K, S_240_T, N_262_T, A_267_S, A281G, K296Q, Q301E, R310G, and V_359_L; for *I.c*, S_57_E, S_59_R, and V_127_L; for *II.a*, T_103_S, K_115_T, F_170_L, E_185_Q, P_186_G, V_189_T, R_310_E, A_315_G, and G_425_A; for *II.a2*, T_103_S, K_115_T, F_170_L, E_185_Q, P_186_G, V_189_T, R_310_E, and G_425_A; for *II.a3*, T_103_S, K_115_T, F_170_L, E_185_Q, P_186_G, V_189_T, R_310_E, A_315_G, and C_420_R; for *II.a4*, T_103_S, K_115_T, F_170_L, E_185_Q, P_186_G, V_189_T, R_310_E, and A_315_G; for *II.b*, T_103_S, K_115_T, and F_170_L; for *II.b2*, D_43_N, T_103_S, K_115_T, and F_170_L; for *II.c*, T_103_S, K_115_T, F_170_L, E_185_Q, P_186_G, V_189_T, R_310_E, A_315_G, and Y_350_STOP; for *III*, W_417_STOP. Download FIG S2, PDF file, 0.2 MB. Copyright © 2019 Pelegrin et al. 2019 Pelegrin et al. This content is distributed under the terms of the Creative Commons Attribution 4.0 International license.

Four different AMR determinants known to confer reduced susceptibility to quinolones were found. The *gyr*A modification that confers resistance to quinolones in P. aeruginosa (through the T83I amino acid substitution) was present in 105/130 (80.8%) strains, all of which belonged to the prevalent ST235, ST357, and ST823 clones and to minor clone ST244. The T83I amino acid substitution in *gyrA* was present in all strains that had MICs of ≥4 mg/liter for ciprofloxacin (Fig. 2B, subplot 3). Two additional amino acid substitutions (H148N and D682E) were found in this gene. Additional mutations were found in genes *gyrB*, *parC*, and *parE* (see Table S4). The aminoglycoside resistome of the collection constituted 21 different aminoglycoside-modifying enzymes and their variants, but the most prevalent aminoglycoside resistant determinant was chromosomally encoded APH(3′)-IIb, which was present in all strains. Additionally, we detected mutations in *mexZ* and *fusA1*, both genes previously linked to aminoglycoside resistance (15) (see Table S4). We observed that all these genes were associated with various levels of susceptibility to amikacin (Fig. 2B, subplot 4). Regarding polymyxin resistance, we did not find any of the plasmid-mediated *mcr* genes. With RGI-CARD, we found only three AMR determinants (*arnA*, *basR*, and *basS*). *arnA* is a component of the *arnT* operon but was the only gene of the operon present. The response regulator gene (*basR*) of the two-component regulatory system BasRS was absent in all strains belonging to the prevalent clones ST357 and ST823 and to the minor clones ST1076, ST312, and ST3277. We expanded this analysis with the mutational resistome, including other genes related to polymyxin resistance. Finally, genes related to bicyclomycin, fosfomycin, and chloramphenicol resistance (*bcr-1*, *fosA*, and *catB*, respectively) were present in all isolates of the collection.

### Genomic epidemiology.

**FIG 3:**
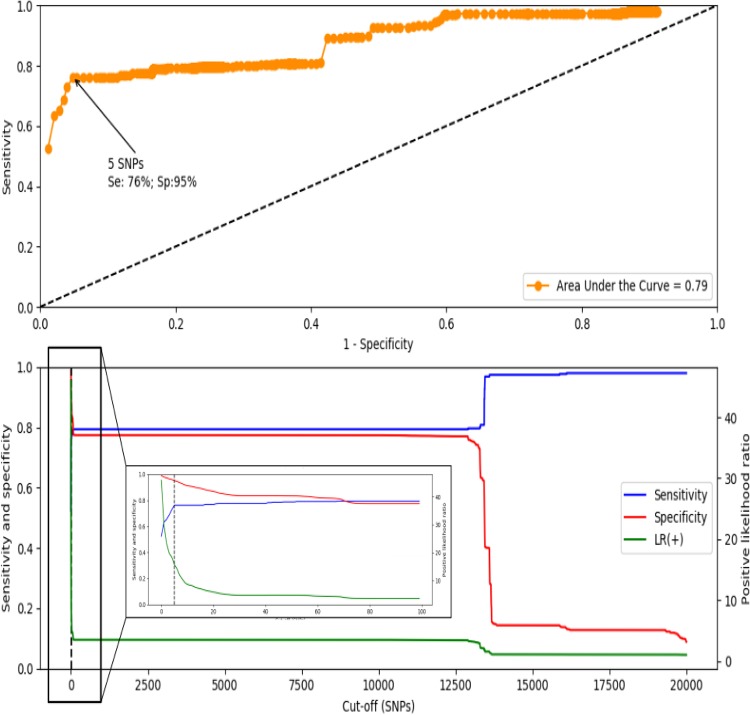
(Top image) Receiving operator characteristics curve. The area under the curve value is represented at the right corner of the image. (Bottom image) Cumulative distribution analysis showing the effects of variations in the cutoff SNP values on sensitivity (blue line), specificity (red line), and the positive likelihood ratio (green line). A vertical dashed black line shows the threshold corresponding to 5 SNP. A zoomed image of the first 100 cutoffs is displayed inside a box.

The optimal threshold for distinguishing isogenic CNPA strains from other strains circulating in this clinical setting was found to be a difference of ≤5 in the number of single nucleotide polymorphisms (SNPs). Thus, isolates that had core genome SNP profile differences below this threshold were considered to belong to the same (i.e., isogenic) strain present in this clinical setting. This cutoff value was associated with *a priori* sensitivity and specificity values of 0.76 and 0.95, respectively (Fig. 3).

**FIG 4:**
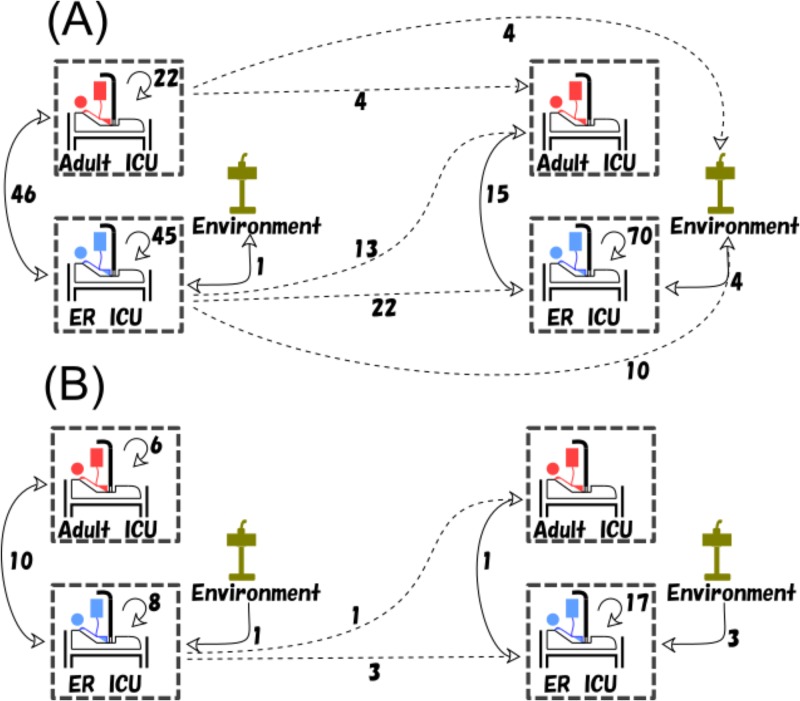
(A) Potential transmission events (PTEs) and (B) acquisition events (AEVs) from known sources during the study period. The boxes consisting of dashed lines represent the two intensive care units, during the preintervention and postintervention phases (panels on the left and right, respectively). Environmental sources of CNPA are depicted by green-colored sinks. Dashed lines represent transmissions (both PTEs and AEVs) between the two study phases; note that such transmissions have been registered as belonging to the postintervention phase.

This cutoff value of 5 SNPs was compatible with the median number of differences in SNPs found between isolates belonging to the dominant multilocus sequence types (MLSTs); these median (range) SNP differences were 59 (0 to 82) for strains belonging to ST235, 17 (0 to 32) for ST357 strains, 5 (0 to 54) for ST446 strains, and 5 (0 to 54) for ST823 strains. We observed several cases where multiple CNPA isolates cultured from the same patient had the same multilocus sequence type but differed by more than the threshold value of 5 SNPs, indicating that they were different strains circulating independently from each other in this clinical setting at the time of the study. That finding included patients with isolates that were acquired in the ICU and patients with isolates that were imported into the ICU. Also, we observed that exclusion of prevalent sequence types from the calculations had some impact on the optimal cutoff value (Table S6), indicating that the genotypic composition of a collection of CNPA isolates may generate (slightly) different optimal cutoff values. Using the 5-SNP threshold, the possible transmission events (PTEs) occurred in the ICU setting at rates of 27.7/100 admissions and 38.0/100 admissions during the preintervention and postintervention phases, respectively. However, the rates of acquisition events (AEVs) were much lower, at 9.2/100 admissions in the preintervention phase and 11.8/100 admissions in the postintervention phase. The majority of AEVs were from known sources. However, sizable minorities of AEVs (38% and 42% in the respective phases of the study) were from an unknown source. Data corresponding to the number of PTEs and AEVs from known sources during the study period are presented in Fig. 4. Most transmission events (PTEs and AEVs) occurred between patients within each of the two ICUs, but such transmissions were also noted to have occurred between the two ICU wards. Environmental sources were mostly linked to transmissions in the ER-ICU, although we registered four PTEs linking adult ICU patients admitted during the preintervention phase to CNPA-positive environmental samples cultured during the postintervention phase, indicating long-term circulation of these strains in the ICU. Using either of the two approaches, we did not find a statistically significant difference between the PTE or AEV rates in the preintervention period versus the postintervention period (*P* values of >0.05). However, the observed number of AEVs in the adult ICU dropped from six to zero in the preintervention period versus the postintervention period. In contrast, the number of AEVs increased from 8 to 17 in the ER-ICU at the same time. Similar contrasting trends were observed while tracing PTEs.

10.1128/mBio.02384-19.8 TABLE S6 Table showing the effect of the genotypic composition of the input samples in the cutoff calculation. “original” indicates the current composition of the collection of carbapenem-nonsusceptible P. aeruginosa isolates. Each “NOT” row represents removal of all isolates of one of the main sequence types described. Download Table S6, DOCX file, 0.01 MB. Copyright © 2019 Pelegrin et al. 2019 Pelegrin et al. This content is distributed under the terms of the Creative Commons Attribution 4.0 International license.

Finally, the calculated Hill numbers for the CPNA isolates collected in the preintervention and postintervention phases were found to have increased (^0^*D*, 20 and 29, respectively; ^1^*D*, ∼10 and ∼20, respectively; ^2^*D*, ∼7 and ∼17, respectively), indicating that the genotypic diversity among the CNPA isolates cultured during the postintervention phase had increased compared to the genotypic diversity of the collection before the intervention.

## DISCUSSION

In this study, carbapenem-nonsusceptible Pseudomonas aeruginosa (CNPA) strains were found to be endemic in the ICUs of the Dr. Cipto Mangunkusumo General Hospital in Jakarta, Indonesia. CNPA clones are present in the ICU environment and are regularly being transmitted to and from patients and their immediate environment. We detected four dominant P. aeruginosa clones (ST235, ST357, ST446, and ST823) which have previously been shown to have spread worldwide and to carry a repertoire of antimicrobial resistance-related genes corresponding to resistance elements ranging from carbapenemases (such as *bla*_IMP_ or *bla*_VIM_) to defective outer membrane porins (see below). We also traced CNPA transmissions using whole-genome sequencing (WGS). To do so, we distinguished isogenic CNPA strains by the use of a new methodology based on the differences in the SNP profiles of their core genomes and on the patients from whom they were cultured. An optimal SNP threshold—below which strains are considered isogenic—was calculated and then applied to trace transmissions of CNPA in this setting. One approach identified potential transmission events based solely on isogenic strains being cultured from different sites. In another approach, we additionally used clinical data to infer CNPA acquisitions by patients during their ICU stay. The latter approach helped us focus on CNPA transmission routes relevant for nosocomial infection control.

We also assessed the impact of an infection control intervention that was implemented in both ICUs to reduce transmissions of multidrug-resistant pathogens. We did observe a large shift in the distribution of CNPA clones, together with an increase in the genotypic diversity of the CNPA population. However, overall, the rates of acquisition of CNPA strains by patients during their ICU stay did not change significantly. Interestingly and inexplicably, a reduced transmission rate in one ICU was accompanied by an increased transmission rate of CNPA in the other ICU. Since the study did not include comparator ICUs that were not intervened with, the observed changes might well be a reflection of the natural variation in the epidemiological dynamics of CNPA in such settings. Although the two ICUs were under same management, the ICUs are in different buildings and have dedicated nursing staffs and also differ in their rates of turnover of patients, all of which might have confounded the effects of the intervention. Either prospective comparative study designs or quasiexperimental designs such as interrupted time series analysis would be needed to come to more-definitive evaluations of the effect of hygienic interventions on the epidemiology of CNPA in intensive care units.

The four main sequence types described in the ICUs of this hospital in Jakarta belong to the so-called P. aeruginosa high-risk international clones reported around the globe that are associated with MDR profiles. Of the four, ST235 and ST357 have been extensively reported in several countries (16–19), while the other two main STs, ST823 and ST446, emerged more recently. A recent study of MDR P. aeruginosa in Malaysia also found ST235, ST357, and ST446 in a hospital setting (20). ST823 was previously described as a minor sequence type found in a multicenter study undertaken in the Gulf Cooperation Council states; more specifically, this clone was found among isolates from Qatar and the United Arab Emirates (21). ST823 has also been found in India and was shown to contain an atypically long genomic island harboring *bla*_VIM-2_ (22). ST446 has been isolated in small clusters or singletons in Spain, The Netherlands, eastern France, and Belgium, showing MDR or carbapenemase-producing profiles (23–26). None of the minor sequence types of CNPA in this study harbored genes coding for carbapenemases, including the ST244 strains, which were linked to the production of *bla*_VIM-2_ carbapenemase in a previous study in West and Central Africa (27). One of the possible consequences of the intervention that we cannot readily explain is the replacement of ST235 by ST357 as the dominant clone. We argue that this substitution may have been the consequence of the intervention, which involved cleaning up environmental sites and limiting transmission initially but was not able to maintain hygienic vigilance over time (28). Alternatively, the waxing and waning of the levels of different CNPA sequence types over time may be the rule rather than the exception in ICUs, and this natural trend may not have been influenced much by the infection control intervention applied in our ICU setting. In addition, we did not observe significant changes in the number and composition of AMR determinants in the two sequence types, which argues against changes in antibiotic pressure having been a direct cause of the replacement of the CNPA strains. Other hypotheses that we contemplated included the possibility of changes in the virulence patterns, as described previously by Bricio-Moreno et al. (29), or of different levels of susceptibility to certain disinfectants such as chlorhexidine, as the use of chlorhexidine-based bathing and mouthwash was part of the intervention.

We wanted to see the full repertoire of AMR genes present in our collection, because P. aeruginosa is well known for its capacity to expand its resistome, especially in hospital settings, with the ICU as an important hot spot (30). In a recent study determining the resistomes of 672 P. aeruginosa strains, Jaillard et al. identified 147 loci associated with antimicrobial resistance, including associations between AMR markers and antibiotics not described before (31). Around 40% of the AMR determinants described in our collection overlapped those described by Jaillard et al. These differences might be explained by the huge diversity in the P. aeruginosa genome; i.e., over 30 different aminoglyoside resistance markers were present, plus mutations in other AMR determinants such as *mexZ* or *fusA1* could contribute to the global aminoglycoside resistance of the strains (15). Predictably, over two-thirds of the genotypically unique strains (87/130, 66.9%) in our CNPA collection carried a carbapenem-degrading enzyme. Back in 2015, Potron et al. (32) published a review focusing on the AMR mechanisms and epidemiology of multidrug-resistant Acinetobacter baumannii and P. aeruginosa which featured several tables that listed all known carbapenemases, including those found in our study. Interestingly, while *bla*_GES-5_, *bla*_IMP1-7-43_, and *bla*_VIM-2_ had been previously reported in Asian countries, including Japan, China, India, and Malaysia, *bla*_VIM-8_ had been previously reported only in Colombia in South America (33). To our knowledge, this study is the first to report this specific *bla*_VIM-8_ type outside South America.

Another well-known mechanism of nonsusceptibility to carbapenems is a defective OprD, an outer membrane porin in P. aeruginosa (34). We found several amino acid substitutions and insertion and/or deletion events in the tertiary structure of the OprD porin protein, but we could not associate them with specific resistance phenotypes. All these point mutations have been previously described (35–37). Specific point mutations (i.e., early stop codons) and frame shifts may be involved in the loss of the OprD porin and, therefore, may affect carbapenem susceptibility (38, 39).

The spread of MDR Gram-negative bacteria carrying carbapenem resistance genes, such as those described above, has been greatly influenced by several factors at the local and global scales, including pathogen and host characteristics, antibiotic prescription practices, and public health policies (40). Furthermore, there is increasing evidence relating antibiotic consumption to the rise of AMR. A recent retrospective study in 153 tertiary hospitals in China significantly correlated the use of carbapenems to the rate of isolation of carbapenem-resistant Gram-negative bacteria, including P. aeruginosa (41). To address this issue, antimicrobial stewardship programs (ASP) aimed at optimizing the use of broad-spectrum antibiotics have been set up in different forms and contexts (42). According to a recent meta-analysis, ASP outcomes translate to smaller amounts of broad-spectrum antibiotics consumed and fewer infections by MDR microorganisms, among other benefits (43). As an example, an ASP to restrict the use of carbapenems was implemented in the ICU of a Saudi Arabian hospital, effectively reducing the prevalence of MDR strains among the P. aeruginosa isolates (44).

Until a few years ago, hospitals from high-income countries relied on techniques such as pulsed-field gel electrophoresis (PFGE) to classify nosocomial pathogens into genetically closely related groups called genotypes, such that their epidemiology could be ascertained. However, WGS provides maximum discriminatory power and is deemed able to unequivocally assign identity to them (45). Two WGS typing approaches have emerged: (i) multilocus sequence typing based on whole/core genomes (wg/cgMLST) and (ii) analysis of single nucleotide polymorphisms across the whole/core genome (wg/cgSNP) (46, 47). Our typing method, based on cgSNPs and clinical data, depends on the quality of each of these data sources. The calculated optimal SNP threshold value depends to some degree on the genotypic composition of the collection of isolates under analysis. We have shown that the diversity of a bacterial population may influence this cutoff value, although it remains fairly similar to the threshold of 4 SNPs established in other studies (11, 48). Thus, there may not be a single optimal SNP cutoff value to distinguish isogenic strains across different collections of P. aeruginosa. However, an optimal SNP cutoff value can be derived for each collection using the new method that we describe in this report.

We would like to point out some other limitations in our study design, the main being the relatively small number of environmental isolates included. Environmental samples were taken only once during each of the two phases of the study. Thus, much more frequent environmental sampling is needed, as was done for the patients themselves, to better detect the niches and transmission routes of CNPA in the intrinsic environment of the ICUs. Similarly, each of the health care personnel was sampled only twice, which may not have been a sufficiently sensitive method to exclude their potential role as a (intermediate) reservoir, source, or vector of CNPA. Another issue is that we did not target carbapenem-susceptible P. aeruginosa (CSPA); doing so would have allowed a deeper understanding of the role of resistance genes in the epidemiology of P. aeruginosa in this health care setting and would have further clarified the development of the resistome of this important nosocomial pathogen. Finally, all our calculations were made using a couple of custom Python scripts and some manual counting. This makes the process not fully automated and, in its present form, not fully scalable. Further steps to improve the scalability and reproducibility of this methodology are necessary.

### Conclusions.

Using whole-genome sequencing in combination with clinical data, we were able to closely track and trace the endemic spread of isogenic carbapenem-nonsusceptible strains of Pseudomonas aeruginosa over a 3-year period in the ICUs of a single tertiary care hospital in Indonesia, a large tropical middle-income country. We observed significant changes in the clonal composition of CNPA and provide insight into the dynamics of transmission of these strains over time but are unable to directly ascribe these changes to the infection control interventions applied. Additionally, we detected the presence of high-risk international clones of multidrug-resistant P. aeruginosa in Indonesia and present their resistomes.

## MATERIALS AND METHODS

### Ethics approval.

The Ethics Committee of the Faculty of Medicine, Universitas Indonesia, approved the research on 17 September 2012 (approval no. 561/PT02.FK/ETIK/2012 and 757/UN2.F1/ETIK/X/2014). Informed consent was documented by the use of a written consent form that was approved by the Ethics Committee Faculty of Medicine Universitas Indonesia/Dr. Cipto Mangunkusumo General Hospital and that was signed and dated by the subjects or their guardians and by the person who conducted the informed consent discussion and two witnesses. The signature confirmed that the consent was based on information that had been understood.

### Study design—sample collection.

We performed a prospective, quasiexperimental before-and-after study in two ICUs of the national referral hospital of Indonesia. Dr. Cipto Mangunkusumo Hospital is a 1,200-bed university hospital located in Jakarta. We conducted this study in two ICUs for adult patients, the 12-bed adult ICU and the 8-bed emergency room ICU (ER-ICU), with averages of 1,010 and 415 admissions per year, respectively. Both ICUs have an open ward design. The populations served by these two ICUs were very similar, and there was also no difference in the care provided (8). The study consisted of three study phases, namely, a preintervention phase (April to October 2013 and April to August 2014), an intervention phase (December 2014 to January 2015), and a postintervention phase (February to December 2015) (8, 28).

CNPA strains were collected from clinical cultures and by targeted screening in the preintervention and postintervention phases. Health care personnel and the ICU environment were screened for CNPA as well (once each in the preintervention and postintervention phases of the study). A list of the isolates, together with clinical data, can be found in Table S5 in the supplemental material. All isolates were stored in 10% glycerol-containing media and were frozen at –80°C until further use. This study has been registered at www.trialregister.nl (no. 5541; candidate no. 23527; Netherlands Trial Register [NTR] trial no. NTR5541; date of NTR registration, 22 December 2015). Further details on the wards for the period 2013 to 2014, the sampling process, and the microbiological methods, such as the CNPA selection criteria, have been previously described in detail (8). Replicate collections of all these isolates were archived in Jakarta (Indonesia), Rotterdam (The Netherlands), and La-Balme-les-Grottes (France).

10.1128/mBio.02384-19.7 TABLE S5 Detailed information regarding the clinical data of the samples and patients included in the study. Download Table S5, XLSX file, 0.1 MB. Copyright © 2019 Pelegrin et al. 2019 Pelegrin et al. This content is distributed under the terms of the Creative Commons Attribution 4.0 International license.

### Intervention.

Between the two collection periods mentioned above, an infection control bundle aimed at reducing transmission of carbapenem-nonsusceptible P. aeruginosa, Klebsiella pneumoniae, and Acinetobacter baumannii-Acinetobacter calcoaceticus complex was implemented in both ICUs. The measures adopted with this intervention included enhanced environmental cleaning, enforced antibiotic stewardship (including daily evaluation of all antibiotic prescriptions on weekdays), and a targeted hand hygiene education for health care workers of the ICUs (28). Once-daily bathing with chlorhexidine 2% was introduced; for intubated patients, oral hygiene procedures were performed four times per day by rinsing with 2% chlorhexidine solutions. Patients colonized or infected with carbapenem-nonsusceptible Gram-negative bacteria were grouped together in a dedicated area of the ward, with contact isolation precautions performed as recommended by the CDC (https://www.cdc.gov/infectioncontrol/basics/transmission-based-precautions.html).

### Bacterial identification, antibiotic susceptibility testing, and DNA extraction.

Stored strains were regrown from the –80°C stocks using Columbia agar plus 5% sheep blood (COS plates; bioMérieux, Marcy-l’Étoile, France) and colonies confirmed to contain Pseudomonas aeruginosa using Vitek MS with standard acquisition parameters according to the instructions of the manufacturer (bioMérieux). Antibiotic susceptibility testing (AST) was performed with Vitek 2 (bioMérieux) using EUCAST 2019 breakpoints (49). The antibiotics tested were ticarcillin, piperacillin, ticarcillin-clavulanic acid, piperacillin-tazobactam, ceftazidime, cefepime, imipenem, meropenem, aztreonam, ciprofloxacin, levofloxacin, amikacin, gentamicin, and tobramycin. Susceptibility to colistin was not reported, since a validated automated test to do so was lacking.

### Whole-genome sequencing and bioinformatics.

DNA was extracted from pure cultures using an UltraClean microbial DNA isolation kit (Qiagen N.V., Venlo, The Netherlands), and quantity and quality were assessed using a Qubit double-stranded DNA (dsDNA) BR assay kit (Thermo Fisher Scientific, Waltham, MA, USA).

### (i) Whole-genome sequencing methods and quality control.

Samples from the preintervention phase were sequenced using either a HiSeq 2500 instrument (Illumina Inc., Cambridge, United Kingdom) with 150-bp paired-end reads or a MiSeq instrument (Illumina Inc.) with 200-bp paired-end reads. Samples from the postintervention phase were sequenced using a NextSeq 500 instrument (Illumina Inc.), with 150-bp paired-end reads. A Nextera XT DNA library preparation kit (Illumina Inc.) was used in all cases. Paired-ended reads were assembled into contigs and scaffolds using the A5-MiSeq pipeline (v20160825) (50). Correct identity of assemblies was confirmed by average nucleotide identity (ANI) analysis using FastANI (v1.2) (51), with P. aeruginosa PAO1 as the reference (GenBank accession no. NC_002516.2). QUAST (v5.0.2) was run to assess the assemblies quality, using standard parameters and with the inclusion of the “–scaffold” parameter when scaffolds were obtained from the assembly (14). Reads and assemblies from all sequenced samples are available at the European Nucleotide Archive website under project identifiers (IDs) PRJEB30625 and PRJEB32907, for the clinical and environmental samples, respectively.

**(ii) Antimicrobial resistance and MLST typing.** Antimicrobial resistance determinants were identified from assemblies using the Resistance Gene Identifier (RGI) command line tool associated with the Comprehensive Antimicrobial Resistance Database (52–54) (updated in 2018; analysis date, December 2018), using the “Strict algorithm,” which allows the detection of previously unknown AMR genes. To display the results obtained using the RGI tool, we used a cluster map created with a custom Python 3 script. Additionally, we screened the literature for genes belonging to the mutational resistome of P. aeruginosa and analyzed them using Snippy (v1.4.1) (Seemann T [2015] snippy: fast bacterial variant calling from NGS reads [https://github.com/tseemann/snippy]) by aligning the contigs to the P. aeruginosa PAO1 reference genome (GenBank accession no. NC_002516.2) and selecting missense variants with a minimum coverage of ×50. BioNumerics 7.6 (Applied Maths, St-Martens-Latem, Belgium) was used for *in silico* multilocus sequence typing (MLST) using the pubMLST database site (hosted at https://pubmlst.org by the University of Oxford). goeBURST was used to infer the relatedness of the MLST profiles (55).

**(iii) Genomic epidemiology of the bacterial strains.** We used the assemblies to perform a *k*-mer-based SNP analysis using kSNP3 (v3.01) (56). kSNP3 was executed with the parameters “–k 21 –core,” setting the *k*-mer nucleotide length to 21 bp and allowing the calculation of the “core SNPs.” The “core SNPs” were those identified from *k*-mers present in all input samples. The goal was to use the SNP data and the available clinical information to infer patterns of transmission of individual CNPA strains between patients during the whole study period. To do so, the first step was to determine a similarity SNP cutoff value below which all isolates would be considered genetically identical, i.e., isogenic. From among all of the kSNP3 output files, we chose the file containing the *k*-mer core SNPs detected (“core_SNP_matrix.fasta”) and used it as the input file for snp-dists (v0.6; https://github.com/tseemann/snp-dists). snp-dists was executed with the “–a –b” parameters. We used the pairwise SNP matrix to evaluate different SNP thresholds. To calculate the optimal SNP threshold value, we assumed that truly identical strains could be cultured only from the same patient and that different patients never shared the same strain. Thus, we considered a true positive (TP) match to have been identified when the number of SNP differences between two isolates from the same patient was below or equal to the tested threshold, a true negative (TN) when the number of SNP differences between two isolates from different patients was above the tested threshold, a false positive (FP) when the number of SNP differences between two isolates from different patients was below or equal to the tested threshold, and a false negative (FN) when the number of SNP differences between two isolates from the same patient was above the tested threshold. Using a custom Python script, we counted the number of true positives (TPs), true negatives (TNs), false positives (FPs), and false negatives (FNs) and then calculated sensitivity and specificity values for a large range of threshold values (0 to 20,000). Sensitivity and specificity values for these different SNP thresholds were used to calculate and plot a cumulative distribution analysis figure and receiver operating characteristic (ROC) curves to finally select the optimal similarity SNP cutoff value using Youden’s index. Isolates that had numbers of SNP profile differences below this cutoff value were considered to be isogenic, i.e., to represent the same strain circulating in this ICU setting at the time of the study. We also evaluated the effect of adjusting the genotype distribution of the CNPA collection on the optimal SNP cutoff value by similarly calculating optimal SNP cutoff values for different subcollections of our initial panel of P. aeruginosa isolates.

**(iv) Possible transmission events versus acquisition events.** In order to highlight the importance of clinical metadata in the outcome and interpretation of the genomic epidemiology analysis, we differentiated between two approaches to account for transmissions of CNPA strains in the ICU setting. The first approach took into account only the genetic concordance between isolates based on SNP differences generated by WGS, yielding what we called “possible transmission events” (PTEs). We defined a PTE as representing the identification of two isolates cultured from two different patients—or from a patient and an environmental sample—that were genetically considered to be the same (isogenic) because the SNP profiles of their core genomes differed by less than the SNP cutoff value (see above). In the second approach, we incorporated both genetic concordance and clinical and other laboratory data, such as patient identifier (ID), date of the culture, and patient admission and discharge dates, to generate what we called acquisition events (AEVs). A patient was considered to have acquired a CNPA in the ICU only when screening at admission was negative and the first CNPA was isolated from a sample taken at least 48 h after admission to the ICU. A patient could have an AEV representing acquisition from either a known or an unknown source. An AEV from a known source was defined as representing an instance in which (i) a patient acquired a CNPA strain that was genetically the same (as defined above) as an isolate cultured earlier from another patient or from an environmental site and (ii) the clinical and microbiological data (e.g., sampling date) could not exclude the possibility that a transmission had occurred between them. If the origin of a CNPA strain cultured from a given patient could not be traced back to a previously identified source, we labeled this AEV “from an unknown source.” To calculate either PTE or AEV, we considered only the first/earliest CNPA strain isolated; subsequent isogenic isolates from the same patient were ignored in enumerating the number of PTEs and AEVs. To compare transmissions before and after the intervention, the occurrence of PTEs and AEVs was expressed as an attack rate, using the total number of patients at risk of CNPA transmission for each period as the denominator. The chi-square test was used to report significance.

Finally, we also calculated the first three Hill numbers (^0^*D*, ^1^*D*, and ^2^*D*) (57) as measures of diversity for the CNPA collections cultured in each of the preintervention and postintervention phases. These Hill numbers represent the following mathematically converted classic diversity indices: ^0^*D*, richness; ^1^*D*, exponent of Shannon-Wiener’s diversity index; ^2^*D*, reciprocal of Gini-Simpson’s index.
