# Erratum for Pelegrin et al., “High-Risk International Clones of Carbapenem-Nonsusceptible Pseudomonas aeruginosa Endemic to Indonesian Intensive Care Units: Impact of a Multifaceted Infection Control Intervention Analyzed at the Genomic Level”

**DOI:** 10.1128/mBio.01372-20

**Published:** 2020-06-16

**Authors:** Andreu Coello Pelegrin, Yulia Rosa Saharman, Aurélien Griffon, Mattia Palmieri, Caroline Mirande, Anis Karuniawati, Rudyanto Sedono, Dita Aditianingsih, Wil H. F. Goessens, Alex van Belkum, Henri A. Verbrugh, Corné H. W. Klaassen, Juliëtte A. Severin

**Affiliations:** a Clinical Unit, bioMérieux, La Balme Les Grottes, France; b Vaccine & Infectious Disease Institute, Laboratory of Medical Microbiology, Faculty of Medicine and Health Sciences, University of Antwerp, Antwerp, Belgium; c Department of Clinical Microbiology, Faculty of Medicine, Universitas Indonesia/Dr. Cipto Mangunkusumo General Hospital, Jakarta, Indonesia; d Department of Medical Microbiology and Infectious Diseases, Erasmus MC University Medical Center, Rotterdam, The Netherlands; e R&D Systems & Development, bioMérieux, Marcy l’Etoile, France; f Microbiology R&D, bioMérieux, La Balme Les Grottes, France; g Critical Care Division, Department of Anesthesia and Intensive Care, Faculty of Medicine, Universitas Indonesia/Dr. Cipto Mangunkusumo General Hospital, Jakarta, Indonesia

## ERRATUM

Volume 10, no. 6, e02384-19, 2019, https://doi.org/10.1128/mBio.02384-19. On PDF page 1, in the abstract, in the sentence “Four high-risk international CNPA clones (ST235, ST823, ST375, and ST446) dominated, but the distribution of these clones changed significantly after the intervention was implemented,” “ST375” within the parentheses should be “ST357.” This change does not change the conclusions of the paper.

