# Supplementary material for: High-Risk International Clones of Carbapenem-Nonsusceptible Pseudomonas aeruginosa Endemic to Indonesian Intensive Care Units: Impact of a Multifaceted Infection Control Intervention Analyzed at the Genomic Level
Source: mBio. 2019 Nov 12;10(6):e02384-19. doi: 10.1128/mBio.02384-19 (PMC6851282; doi:10.1128/mBio.02384-19)
Supplement: FIG S1 [file mBio.02384-19-sf001.pdf]

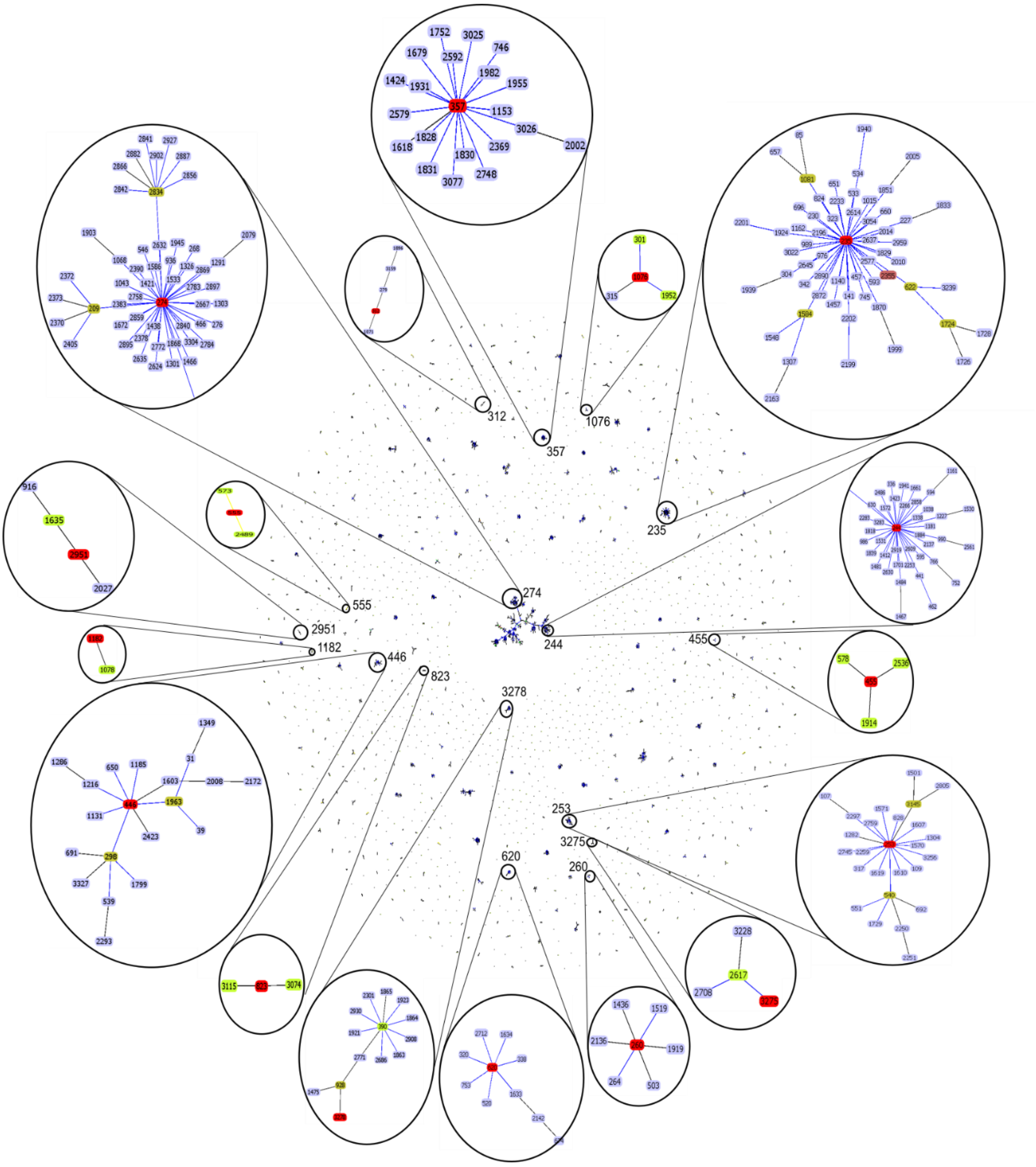

Figure S1. goeBURST of the 3321 sequence types listed in the *Pseudomonas aeruginosa* PubMLST database (August 2019). Blue points represent sequence types (STs); lines connect single-locus variants (SLVs). Red boxes within the circles are the STs found in this study, light green boxes represent the clonal complex group founder, while dark green boxes the sub-group founder. ST1189 and ST3277 are singletons.
