# Supplementary material for: High-Risk International Clones of Carbapenem-Nonsusceptible Pseudomonas aeruginosa Endemic to Indonesian Intensive Care Units: Impact of a Multifaceted Infection Control Intervention Analyzed at the Genomic Level
Source: mBio. 2019 Nov 12;10(6):e02384-19. doi: 10.1128/mBio.02384-19 (PMC6851282; doi:10.1128/mBio.02384-19)
Supplement: TABLE S3 [file mBio.02384-19-st003.doc]

| Sequence type | Number (%) of CNPA | | | Genotype-corrected number (%) of CNPA | | |
| --- | --- | --- | --- | --- | --- | --- |
|  | Pre-intervention (n= 119) | Post-intervention (n= 118) | All (n = 237) | Pre-intervention (n= 64) | Post-intervention (n= 66) | All (n = 130) |
| 235 | 45 (37.8) | 28 (23.7) | 73 (30.8) | 23 (35.9) | 17 (25.8) | 40 (30.8) |
| 823 | 36 (30.3) | 11 (9.3) | 47 (19.8) | 20 (31.3) | 6 (9.1) | 26 (20.0) |
| 446 | 17 (14.3) | 2 (1.7) | 19 (8.0) | 6 (9.4) | 2 (3.0) | 8 (6.2) |
| 357 | 10 (8.4) | 62 (52.5) | 72 (30.4) | 5 (7.8) | 32 (48.5) | 37 (28.5) |
| 1076 | 0 (0) | 4 (3.4) | 4 (1.7) | 0 (0) | 2 (3.0) | 2 (1.5) |
| 244 | 2 (1.7) | 1 (0.8) | 3 (1.3) | 2 (3.1) | 1 (1.5) | 3 (2.3) |
| 455 | 0 (0) | 3 (2.5) | 3 (1.3) | 0 (0) | 1 (1.5) | 1 (0.8) |
| 555 | 0 (0) | 2 (1.7) | 2 (0.8) | 0 (0) | 1 (1.5) | 1 (0.8) |
| 620 | 2 (1.7) | 0 (0) | 2 (0.8) | 2 (3.1) | 0 (0) | 2 (1.5) |
| 1189 | 2 (1.7) | 0 (0) | 2 (0.8) | 1 (1.6) | 0 (0) | 1 (0.8) |
| 260 | 0 (0) | 1 (0.8) | 2 (0.8) | 0 (0) | 1 (1.5) | 1 (0.8) |
| 274 | 1 (0.8) | 0 (0) | 1 (0.4) | 1 (1.6) | 0 (0) | 1 (0.8) |
| 253 | 1 (0.8) | 0 (0) | 1 (0.4) | 1 (1.6) | 0 (0) | 1 (0.8) |
| 1182 | 1 (0.8) | 0 (0) | 1 (0.4) | 1 (1.6) | 0 (0) | 1 (0.8) |
| 2951 | 1 (0.8) | 0 (0) | 1 (0.4) | 1 (1.6) | 0 (0) | 1 (0.8) |
| 312 | 0 (0) | 1 (0.8) | 1 (0.4) | 0 (0) | 1 (1.5) | 1 (0.8) |
| 3275 | 0 (0) | 2 (1.7) | 2 (0.8) | 0 (0) | 1 (1.5) | 1 (0.8) |
| 3277 | 1 (0.8) | 0 (0) | 1 (0.4) | 1 (1.6) | 0 (0) | 1 (0.8) |
| 3278 | 0 (0) | 1 (0.8) | 1 (0.4) | 0 (0) | 1 (1.5) | 1 (0.8) |

**Table S3. Multi locus sequence type frequency of CNPA isolates and genotype-corrected CNPA.**
