# Supplementary material for: High-Risk International Clones of Carbapenem-Nonsusceptible Pseudomonas aeruginosa Endemic to Indonesian Intensive Care Units: Impact of a Multifaceted Infection Control Intervention Analyzed at the Genomic Level
Source: mBio. 2019 Nov 12;10(6):e02384-19. doi: 10.1128/mBio.02384-19 (PMC6851282; doi:10.1128/mBio.02384-19)
Supplement: FIG S2 [file mBio.02384-19-sf002.pdf]

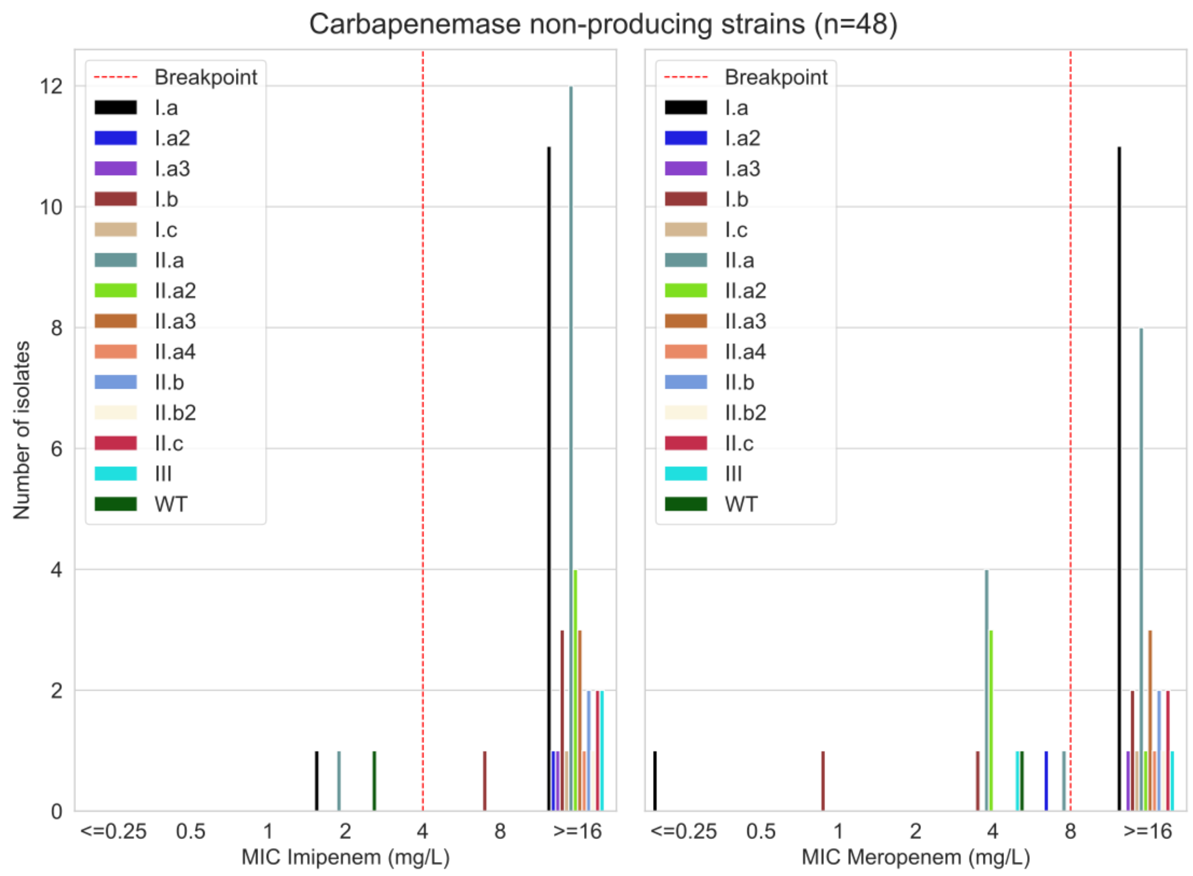

Figure S2. OprD types among the carbapenemase non-producing strains. Three main OprD types and 11 other sub-types were observed. The point mutation pattern is described below, note that sub-types *I.a*, *I.a2*, and *I.a3* additionally carried the $_{372}$(VDSSSS-YAGL-)$_{383}$ indel in the C-terminal part of OprD, firstly described by Epp, *et al*. [40].

- *I.a* ($S_{57}E$, $S_{59}R$, $V_{127}L$, $E_{185}Q$, $P_{186}G$, $V_{189}T$, $E_{202}Q$, $I_{210}A$, $E_{230}K$, $S_{240}T$, $N_{262}T$, $T_{276}A$, $A_{281}G$, $K_{296}Q$, $Q_{301}E$, $R_{310}E$, $A_{315}G$, $L_{347}M$, $S_{403}A$, $Q_{424}E$);
- *I.a2* ($V_{127}L$, $E_{185}Q$, $P_{186}G$, $V_{189}T$, $E_{202}Q$, $I_{210}A$, $E_{230}K$, $S_{240}T$, $N_{262}T$, $T_{276}A$, $A_{281}G$, $K_{296}Q$, $Q_{301}E$, $R_{310}E$, $A_{315}G$, $L_{347}M$, $S_{403}A$, $Q_{424}E$);
- *I.a3* ($S_{57}E$, $S_{59}R$, $V_{127}L$, $E_{185}Q$, $P_{186}G$, $V_{189}T$, $E_{202}Q$, $I_{210}A$, $E_{230}K$, $S_{240}T$, $N_{262}T$, $T_{276}A$, $A_{281}G$, $K_{296}Q$, $Q_{301}E$, $R_{310}E$, $A_{315}G$, **$V_{352}L$**, $S_{403}A$, $Q_{424}E$).
- *I.b* ($D_{43}N$, $S_{57}E$, $S_{59}R$, $V_{127}L$, $E_{185}Q$, $P_{186}G$, $V_{189}T$, $E_{202}Q$, $I_{210}A$, $E_{230}K$, $S_{240}T$, $N_{262}T$, **$A_{267}S$**, A281G, K296Q, Q301E, R310G, **$V_{359}L$**);
- *I.c* ($S_{57}E$, $S_{59}R$, $V_{127}L$);
- *II.a* $T_{103}S$, $K_{115}T$, $F_{170}L$, $E_{185}Q$, $P_{186}G$, $V_{189}T$, $R_{310}E$, $A_{315}G$, $G_{425}A$
- *II.a2* $T_{103}S$, $K_{115}T$, $F_{170}L$, $E_{185}Q$, $P_{186}G$, $V_{189}T$, $R_{310}E$, $G_{425}A$
- *II.a3* $T_{103}S$, $K_{115}T$, $F_{170}L$, $E_{185}Q$, $P_{186}G$, $V_{189}T$, $R_{310}E$, $A_{315}G$, **$C_{420}R$**

- *II.a4* $T_{103}S$, $K_{115}T$, $F_{170}L$, $E_{185}Q$, $P_{186}G$, $V_{189}T$, $R_{310}E$, $A_{315}G$
- *II.b* $T_{103}S$, $K_{115}T$, $F_{170}L$
- *II.b2* $D_{43}N$, $T_{103}S$, $K_{115}T$, $F_{170}L$
- *II.c* $T_{103}S$, $K_{115}T$, $F_{170}L$, $E_{185}Q$, $P_{186}G$, $V_{189}T$, $R_{310}E$, $A_{315}G$, $Y_{350}STOP$
- *III* $W_{417}STOP$
