# Supplementary material for: High-Risk International Clones of Carbapenem-Nonsusceptible Pseudomonas aeruginosa Endemic to Indonesian Intensive Care Units: Impact of a Multifaceted Infection Control Intervention Analyzed at the Genomic Level
Source: mBio. 2019 Nov 12;10(6):e02384-19. doi: 10.1128/mBio.02384-19 (PMC6851282; doi:10.1128/mBio.02384-19)
Supplement: TABLE S6 [file mBio.02384-19-st006.docx]

**TABLE S6.** Effect of the genotypic composition of the collection of Carbapenem non-susceptible *P. aeruginosa* (CNPA) on the optimal SNP cutt-off value to distinguish individual strains.

| **Collection of CNPA** | | **Number of isolates** | **Cut-off**  **(≤ SNP differences)** | **Sensitivity (%)** | **Specificity (%)** |  |
| --- | --- | --- | --- | --- | --- | --- |
| Original | | 237 | 5 | 76.22 | 95.16 |  |
| ^a^ NOT ST235 | | 164 | 3 | 68.27 | 94.69 |  |
| ^a^ NOT ST357 | | 165 | 4 | 75.81 | 94.50 |  |
| ^a^ NOT ST823 | | 190 | 4 | 60.00 | 95.94 |  |
| ^a^ NOT ST446 | | 218 | 4 | 54.85 | 95.23 |  |
| ^a^ NOT ST235&ST357 | | 92 | 3 | 79.65 | 91.19 |  |
| ^a^ NOT ST235&ST357&ST823 | | 45 | 2 | 96.55 | 97.64 |  |
| ^a^ NOT ST235&ST357&ST823&ST446 | | 26 | 61 | 100.00 | 98.73 |  |
|  | ^a^ excluding isolates belonging to the indicated multi locus sequence type. | | | | | |
